# Supplementary material for: Mechanism of Antitumor Effects of Saffron in Human Prostate Cancer Cells
Source: Nutrients. 2023 Dec 28;16(1):114. doi: 10.3390/nu16010114 (PMC10780623; doi:10.3390/nu16010114)
Supplement: Supplementary file 1 [file nutrients-16-00114-s001.zip › Supplementary Table S1.pdf]

Table S1: Primer sequence information.

	Forward Primer	Reverse Primer	TaqMan Probe Sequence
**Primer sequence for RT-PCR**			
β-actin	5’-AGTCCCTTGCCATCCTAAAAG-3’	5’-CAATGCTATCACCTCCCCTG-3’	5’-CCAGTCCTCTCCCAAGTCCACAC-3’
p53	5’-CTCCTCAGCATCTTATCCGAGTG-3’	5’-GGTGGTACAGTCAGAGCCAAC-3’	5’-AGGCGGCTCATAGGGCACCACCA-3’
MST1R	5’-GCGTAGATGGTGAATGTCATATCC-3’	5’-GCTGAAGACCAGTGCAGTCG-3’	5’-CCACAAGCAGCAGCAAAGGCAGCA-3’
WDR70	5’-CAGTGTCTGCTTTGGGTCTGG-3’	5’-TCACAGGGCTGAAGGGATCG-3	5’-CCCTCAGGTGCCCGTTTGGTGACA-3’
RECQ5	5’-GGCAGCTTCATCCTCCTTCC-3’	5’-TCATCCACCACCAAGTAAGACAG-3’	5’-CCCTGCCCTCCCTGGTGTCCCGCC-3’
WRN	5’-GCCTCTCATGACAATTGGCATG-3’	5’-GAGTTGGACGGGAGGGTTTCG-3’	5’-CCCAAGCGGTGAAAGCTGGCTGCC-3’
SIRT1	5′-CCTGTGAAAGTGATGAGGAGGATAG-3	5′-TTGGATTCCCGCAACCTG-3	5’-TCACCACCTAACCTATGACAC-3’
EHMT2	5’-TCAAAACCGCTTTCCTGG-3’	5’-TGTCCCAATGGTCAGCA-3’	5’-AGTGTCCATGCTACCTGGCTGT-3’
MGMT	5’-AAAGGTACGGGCCATTTGG-3’	5’-GGCGCCTTCCCAGCTT-3’	5’- TAAGGCACAGAGAGCCTC-3’

Supplementary Table S1

COMT	5′-CTACTGGCTGACAACGTGATCTG-3’	5′-GTATTCCAGGAACGATTGGTAGTGT-3’	5′-TGCGCCAGACTTCCTAGCACACGTG-3’
**Primer sequence for standard PCR**			
BCL-2	5’-ATGTGTGTGGAGAGCGTCAACC-3’	5’-TGAGCAGAGTCTTCAGAGACAGCC-3’	
IL-2	5’-CGCATCCTTAAGCAGCAACC-3’	5’-CGCATCCTTAAGCAGCAACC-3’	
β-actin	5’-AGTCCCTTGCCATCCTAAAAG-3’	5’-CAATGCTATCACCTCCCCTG-3’	
